# Building sustainable QI capacity through a regional QI education programme: lessons from 8 years of experience with London paediatric resident doctors

**DOI:** 10.3389/frhs.2026.1784992

**Published:** 2026-10-12

**Authors:** Jane Runnacles, Nicola Davey, Emelia Shead, Emma Parish, Alice Roueché

**Affiliations:** 1 London Specialty School of Paediatrics (NHS), London, United Kingdom; 2 St. George’s University Hospitals NHS Foundation Trust, London, United Kingdom; 3 Quality Improvement Clinic, Southampton, United Kingdom; 4 City St George’s University of London - Tooting Campus, London, United Kingdom; 5 Evelina London Children’s Hospital, London, United Kingdom; 6 King’s College London, London, United Kingdom

**Keywords:** faculty development, mentorship, postgraduate education and training, quality improvement, quality improvement education

## Abstract

**Introduction:**

Since 2018, the NHS London Specialty School of Paediatrics has collaborated with QIClearn (an independent organisation) to design and deliver a structured Quality Improvement (QI) education programme for postgraduate resident doctors in the region to build QI capacity. The programme (QI Change Champions) has been designed not only to equip resident doctors with core improvement skills but, uniquely, to secure its own future through a novel, scalable approach to faculty and mentor development. This paper outlines the experience and learning from this programme, discusses transferable lessons and the potential contribution to the wider field of QI education.

**Method:**

We believe we have developed an innovative and sustainable model for embedding essential QI capability into postgraduate training for resident doctors in a region. We have observed QI in practice by constantly reflecting, learning and adapting our approach. Although this is not a formal programme evaluation, we have described our work in practice and the themes captured from mentor and learner feedback used to continually improve the programme. The analysis was designed to mitigate bias, in view of the fact that QIClearn was receiving payment as a company, and the faculty who co-designed the programme were educators for the London Specialty School of Paediatrics and not employed by QIClearn.

**Results:**

We have enrolled 254 resident doctors across 16 iterations (2 per year) to equip them with the skills to deliver meaningful change in the NHS. We recognised early on that sustainable QI education depends on development of a faculty development pipeline. We have trained 14 mentors selected from those who completed the programme who demonstrate particular skill in motivating others and understanding of QI methodology.

**Discussion:**

Our novel Mentor in Training model has enabled us to both expand our regional QI capacity and develop the next generation of clinical leaders and educators. We have built on the growing field of evidence for near-peer mentors in medical education and developed a real community of practice to support our learners. We suggest that there are long term benefits to building this community but this is an area that needs to be explored further in future research..

## Introduction

Despite widespread recognition that Quality Improvement (QI) training should be embedded throughout healthcare professional education, there remains a significant gap between policy recommendations and effective implementation within postgraduate medical training, as discussed further below. Trainees often face competing clinical and educational demands, limited protected time, frequent rotations that disrupt project continuity, and a tendency to engage in QI activities without a deep understanding of improvement methodology. In addition, there is a shortage of experienced QI mentors and supervisors, alongside organisational and hierarchical barriers that limit meaningful trainee engagement in improvement work. While mentorship is known to be a key factor in the success and sustainability of QI projects, there is a particular lack of evidence and structured approaches for developing QI mentors from within the resident doctor workforce. Existing QI communities of practice provide learning opportunities, but formalised near-peer mentoring models are not well established.

The NHS London Speciality School of Paediatrics (LSP) focuses on regional postgraduate training for paediatric doctors (NHS training scheme), including leadership development in QI. Our programme was launched in response to a regional survey conducted by the LSP Resident doctor's Committee showing that resident doctors had variable exposure to QI in their local training units and few had access to mentors experienced in QI methodology. There was a clear opportunity for a more structured QI education programme across the region with allocated mentors and flexibility around training rotations. The LSP has collaborated with QIClearn to design and deliver a structured Quality Improvement (QI) education programme for postgraduate resident doctors in the region to build QI capacity over 8 years. QIClearn is an independent company that designs and provides QI teaching and support in UK healthcare systems with a strong grounding in NHS practice.

This paper presents some of our experiences of this educational innovation. It utilises a longitudinal descriptive design based on routinely collected platform analytics, participation records, and follow-up feedback from 2018 to 2026. This covers 16 iterations of the programme. We surveyed residents and mentors on the programme through formal online surveys at the start and end of the programme and information feedback throughout and during co-production. We hope sharing our learning and observations will inform future research and more rigorous studies into sustainable QI education for frontline clinicians.

### Rationale and vision for a sustainable QI education programme

Over the last decade there has been an increasing international consensus that Quality Improvement training should be a fundamental requirement for all health professionals (1). Following the Mid Staffordshire enquiry in the UK, the Berwick Report recommended, “*Fostering whole-heartedly the growth and development of all staff, including their ability and support to improve the processes in which they work”* (2)*.* The benefit of teaching improvement methodology at an early stage in medical training has since been acknowledged by the UK General Medical Council (GMC) in their revised undergraduate curriculum (3), and in US post-graduate education by the Accreditation Council for Graduate Medical Education (ACGME) (4). The World Federation for Medical Education (WFME) also produced global standards for quality improvement in 2015 (5) where there is a suggestion of the need for academic mentors as part of student counselling. The UK Academy of Royal Medical Colleges Report “QI: Training for better outcomes 2016” also recommends that a ‘progressive curriculum in QI activity should underpin all training stages of a doctor, building capability and leadership’ (6). The Royal College of Paediatrics and Child Health (RCPCH) has delivered QI education through collaboratives and acknowledged QI as one of the eight domains of its revised Progress curriculum (7). Similar recommendations have been made for allied health professionals and nurses internationally (8, 9). Despite this growing body of support, effective integration of QI training into postgraduate medical training remains a significant challenge, which we explore below.

### Challenges of integrating QI into postgraduate training

The implementation of Quality Improvement (QI) education in postgraduate medical training is notoriously difficult due to competing demands on the learner's time (10). Resident doctors face high clinical workloads, limited study leave, and the pressure of professional examinations. Furthermore, projects often suffer from “project churn” due to frequent rotations; resident doctors may refer to “PDSA cycles” without a deep understanding of the Model for Improvement, often leaping to solutions before sufficiently analysing the problem. A significant barrier remains the lack of trained mentors and supervisors with practical experience in QI methodology (10, 11). Marsden et al. (12) also identify hierarchical culture as well as insufficient access to experienced mentors as key barriers that prevent postgraduate trainees from meaningfully engaging in QI activity. These challenges are echoed across the wider workforce: nursing literature similarly demonstrates that sustaining QI initiatives requires ongoing infrastructure, support and protected time (13).

### Theory of near-peer mentorship and communities of practice

To address these challenges, our programme utilizes the concept of “Communities of Practice,” where learning is situated within a social and professional context (14). Stanford et al. (15) emphasise the importance of building self-sustaining communities of practice within training programmes, highlighting that lasting QI capability depends on distributed expertise and structured opportunities for supported project work, in addition to institutional commitment. While communities of practice for QI exist, such as the IHI Open School (16), formal mentoring within medical education for QI is less established. We build upon the growing evidence for near-peer mentorship (17), where those who have recently faced similar clinical and educational challenges provide support to new learners.

### Evidence for mentorship in QI success and sustainability

Mentoring is a recognised component of development in medical leadership and management. Existing literature indicates that structured mentorship significantly impacts the successful implementation of QI projects (10–17). However, there is a distinct gap in the literature regarding how to identify and develop effective QI mentors from within the resident doctor workforce. Sustainable QI education depends on a robust faculty development pipeline. By identifying “QI Change Champions” and transitioning them into “Mentors in Training” (MiT), programmes can create a self-sustaining cycle that builds regional QI capacity while developing the next generation of clinical leaders.

### Ethics and governance

As this work describes the development of a regional postgraduate training initiative, it is classified as an educational innovation rather than human participant research. Consequently, formal institutional ethics board approval was not required. All data presented were derived from routinely collected programme analytics and voluntary feedback surveys conducted as part of the programme's QI process. Programme learners consented to this data collection and analysis when enrolling on the programme, with a view to continually improving the content and design. We maintained participant confidentiality through the use of anonymised datasets at the point of analysis.

### Conflict of interest and bias mitigation

The authors acknowledge a professional collaboration between the LSP and QIClearn, an independent social enterprise that receives funding for the provision of the learning platform and support services. To mitigate potential bias and ensure independent oversight, the approach to understanding the programme’s impact and the analysis of learner data were led by the clinical faculty, who are educators for LSP and not employees of QIClearn. The conclusions drawn represent the observations of the clinical educators involved in the programme's delivery. These educators are NHS employers who have medical education roles (educational supervisors and training programme directors) under the London Specialty School of Paediatrics, the overseeing institution.

### Structure of the programme

The aim of the “QI Change Champions” programme is to equip learners with the skills and habits of an improver (18) whilst working on a local service improvement project in the healthcare setting in which they work. The programme demonstrates how QI is fundamental, and not an add-on, to their role as frontline clinical staff.

The vision is to build regional capacity in QI by developing the learners as future clinical leaders in healthcare. Many become supervisors to resident doctors in the future and indeed leaders in their own department (19), so will be better placed to mentor, teach and support others in QI activities.

Standalone QI workshops have been delivered since 2012 with a formal structured “QI Change Champions” programme piloted in 2017, running bi-annually from 2018 (see Figure 1). From 2020 taught sessions have been extended and delivered virtually, with interactive learning activities replicated to deliver similar, and sometimes enhanced, learning.

**Figure 1:**
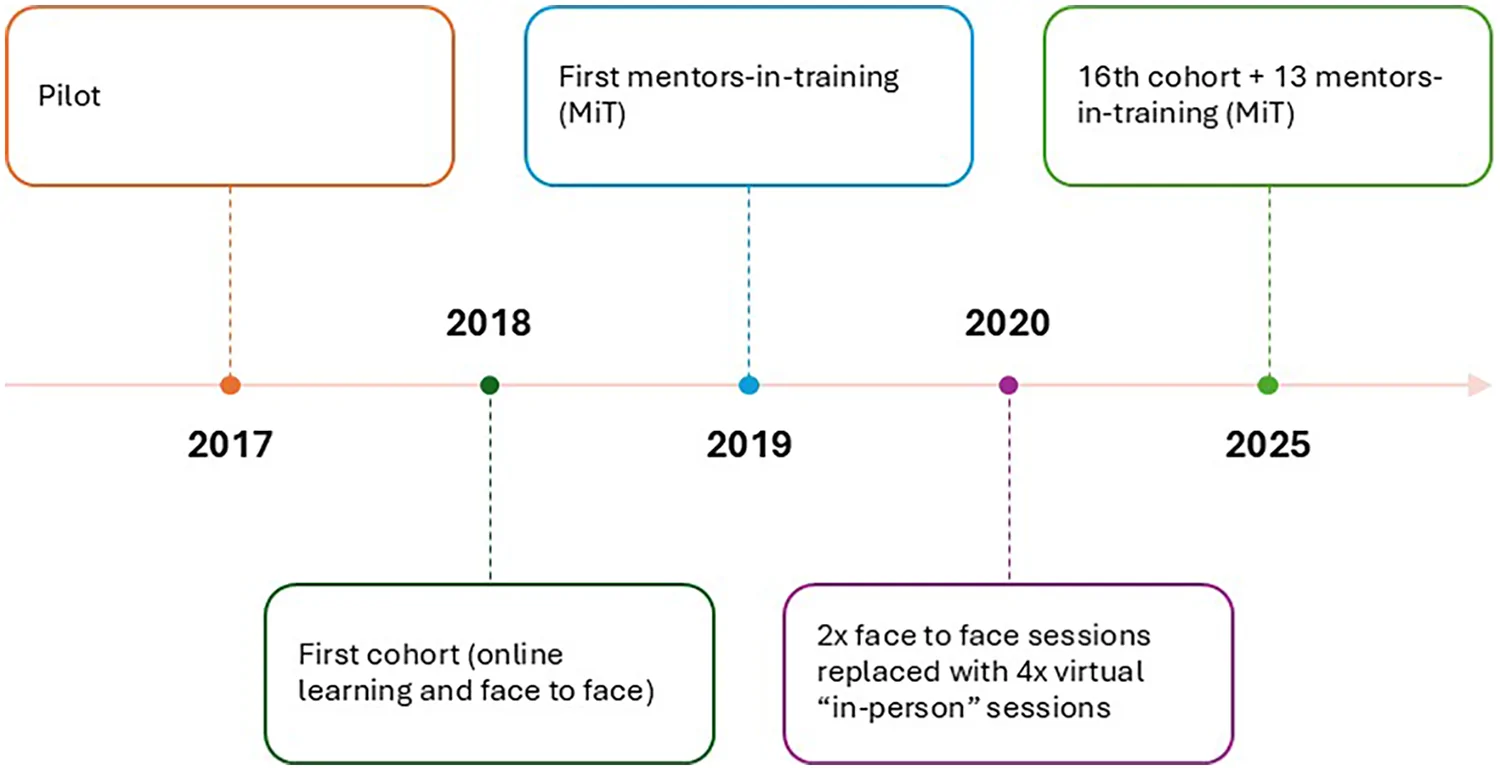
Timeline to illustrate the evolution of the LSP regional QI education programme.

The LSP receives funding from NHS England for postgraduate training and each resident doctor on a training programme is allocated £1,000 for training courses. The collaboration between LSP and QIClearn means that the LSP resident doctors can be fully funded to attend the course. Parents and patients are offered free places.

The programme, has two mandatory full day taught sessions (workshops) and two supplementary virtual sessions, all supported by a bespoke online platform. It links resident doctors with a named mentor and small group learning set to support them in identifying and sustaining a project which can be delivered within the 4 months of the programme (see Figure 2). The average rotation in paediatric training is 6 months and the aim is to equip those who complete the programme to manage future projects in a similar timescale with sound QI methodology.

**Figure 2:**
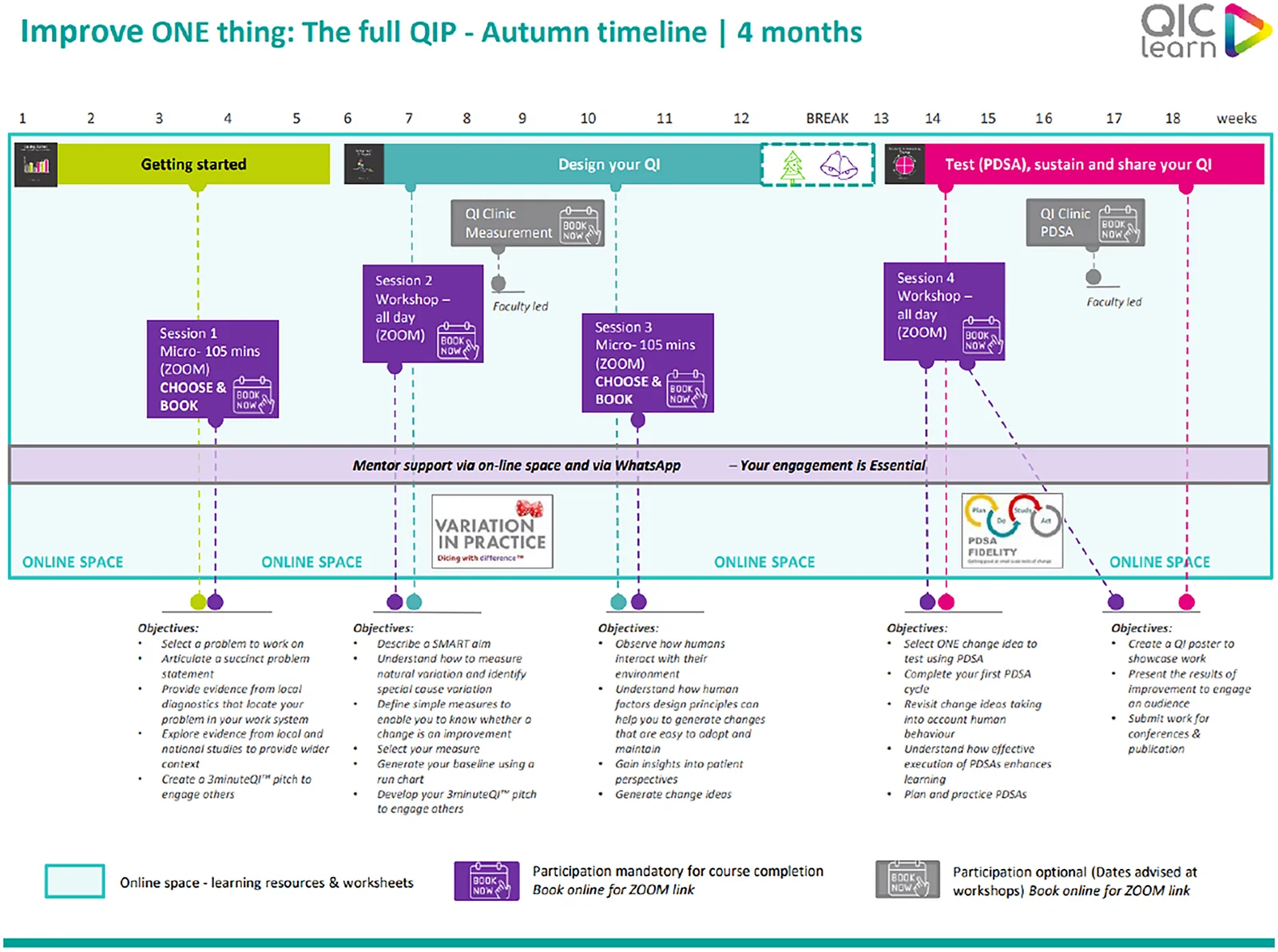
Timeline showing the structure of the 4 month QI education programme.

The online platform, available on mobile devices, includes videos and short teaching pieces to introduce key QI concepts. Alongside these are tools and templates for learners to download and complete. They can then be uploaded for review and comments by mentors and other learners. This enables all learners to contribute and benefit from the feedback for the whole cohort. Gamification with completion targets, leaderboards and “experience points” ensures completion of each level and offers an important opportunity for social learning before progressing further. At the end of the course the online platform generates a slide deck and poster, allowing the uploaded work to be easily presented and shared.

Alongside the online learning, learners are split into small learning sets, each with an assigned mentor. Each set has a maximum of 5 programme learners. These learning sets have additional webinars, informal online chats and ad-hoc meetings for further mentoring and support. There is a smaller faculty group of 5 experienced mentors who deliver the teaching within the workshops and who direct future improvements to the programme. This group consists of four physicians with specialist QI experience (on average 5 + years) and a QI expert and former pharmacist. The mentors have access to metrics to show how their mentees are progressing in the online space and can use this to offer targeted support where needed.

The programme faculty utilise QI methodology to continually review and adapt the structure, approach and content of the teaching materials and learning opportunities. The experience to date has been in both adapting the programme to a blended online and virtual in-person delivery, as well as reflecting on the qualities of what makes a good educational mentor and the optimum contact points with learners (for example, a mentor briefing within 2 weeks of the start to discuss their chosen problem).

### Progressive achievement

At the end of the programme learners are awarded one of three levels of professional development (Figure 3), depending on their engagement with the programme:

1. Certificate of completion - for attendance at virtual taught sessions only.
2. QI Role Model- attendance at virtual taught sessions and progress in an individual project, demonstrating an understanding of QI methodology including measurement for improvement.
3. QI Change Champion- attendance at virtual taught sessions and completion of a QI project using structured change methodology, including testing of ideas (several PDSA cycles) and run chart with learning and reflection.

**Figure 3:**
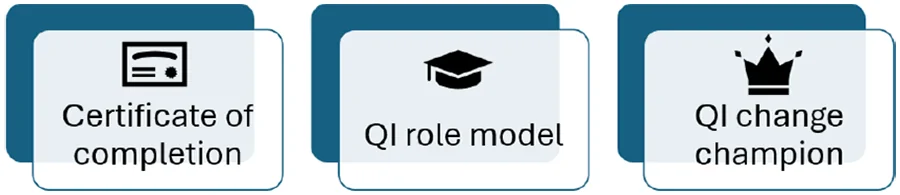
The three levels of programme achievement.

There has been an overall increase in completion rates over time, with more resident doctors progressing to become QI change champions (Figure 4).

**Figure 4:**
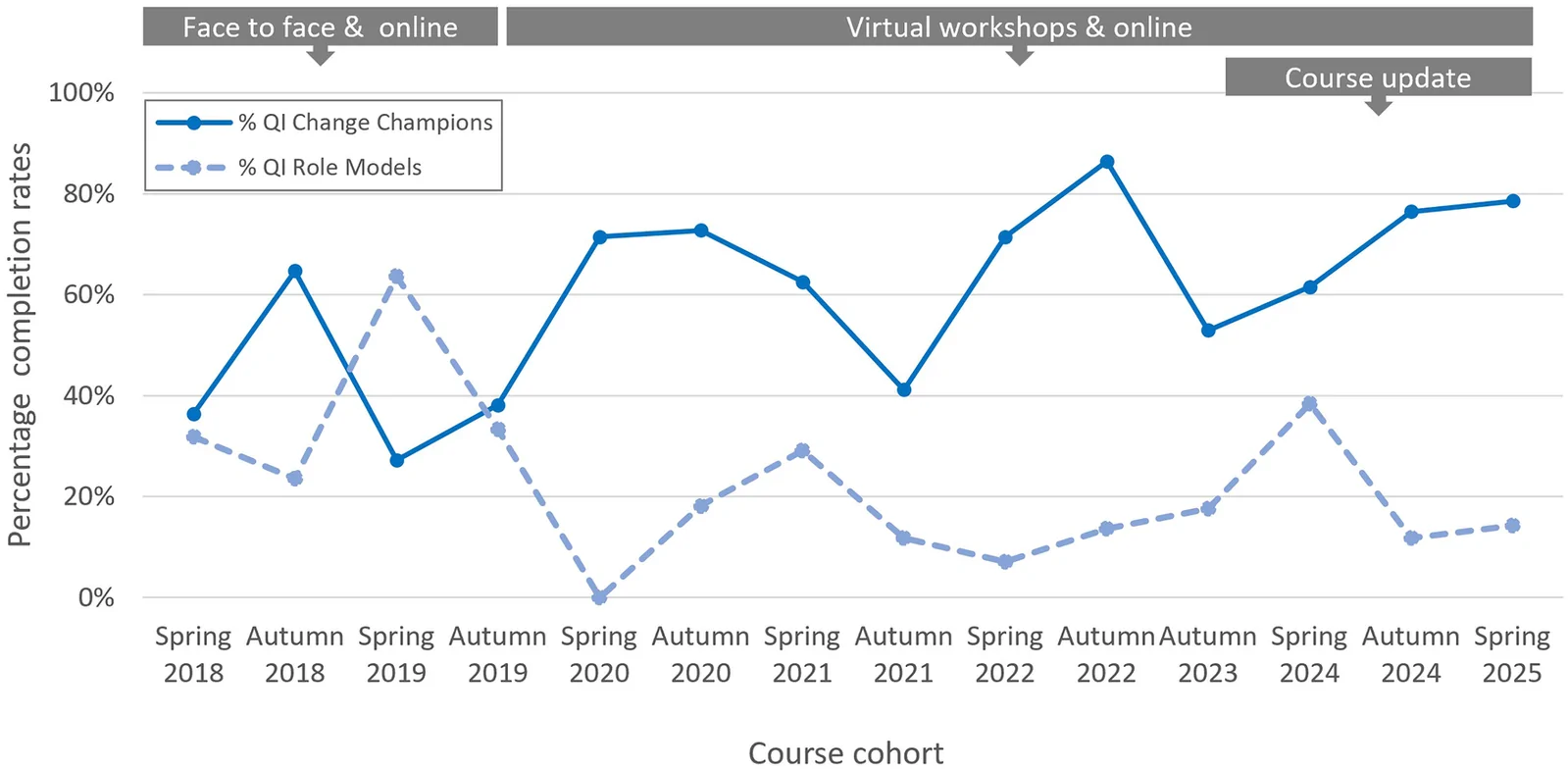
Completion rates of the programme demonstrating percentage of QI role models and QI champion champions by course cohort.

QI Change Champions who show an interest in becoming a mentor, and who are recognised as providing supportive and constructive feedback to their peers, are invited to return to a subsequent programme as a “Mentor in Training” (MIT) to gain further experience in QI whilst supporting new learners alongside a more experienced mentor. This offers a unique approach to faculty development in QI education to ensure sustainability in programme delivery and additional benefits for resident doctors to develop their leadership skills.

### Improvement in context

The importance of developing “habits of an improver” (18) is well recognised by the faculty. Since the resident doctors are time-limited, it is important to ensure their aims are small and achievable. The programme has been continually adapted using QI methodology in response to feedback from both learners and mentors to ensure the quality of the QI projects is high (outlined in Figure 5).

**Figure 5:**
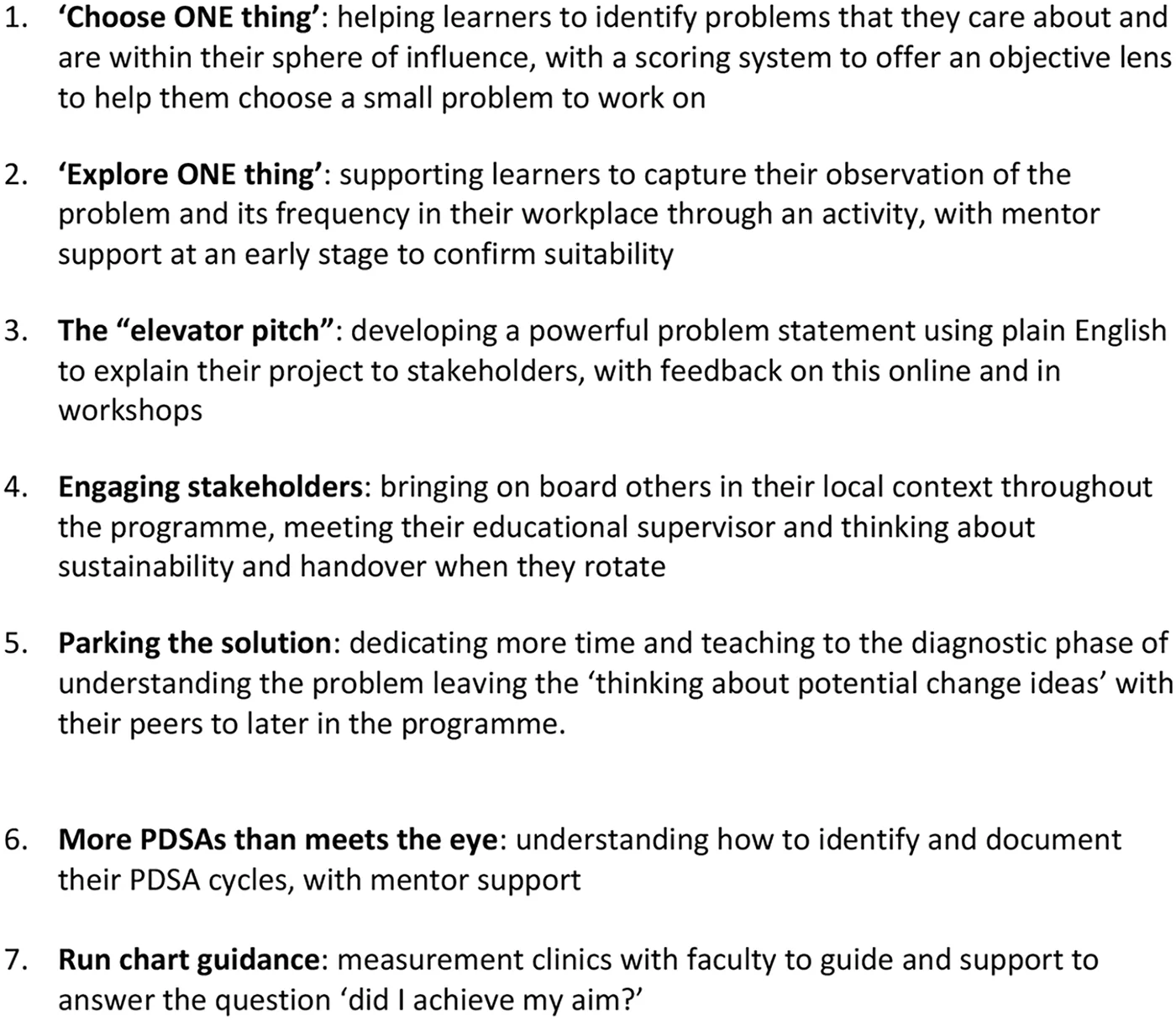
Examples of key learning in the programme design.

Programme learners are encouraged to meet with their local educational supervisors, consider their local stakeholders and access existing resources, with local sign posting and support. This is crucial both for the success of their local QI project but also to gain further support for the programme, encouraging their supervisors to promote the programme to other resident doctors who rotate through their department. A common pitfall seen through our experience are learners coming with QI projects which are solution-based rather than problem-focussed. We are able to support both resident doctors and their local mentors in navigating this. In this way, the programme mentors do not replace local mentors but rather improve the access learners have to those with QI expertise.

The feedback from learners suggests that mentorship is crucial to their learning on this programme (see Figure 6).

**Figure 6:**
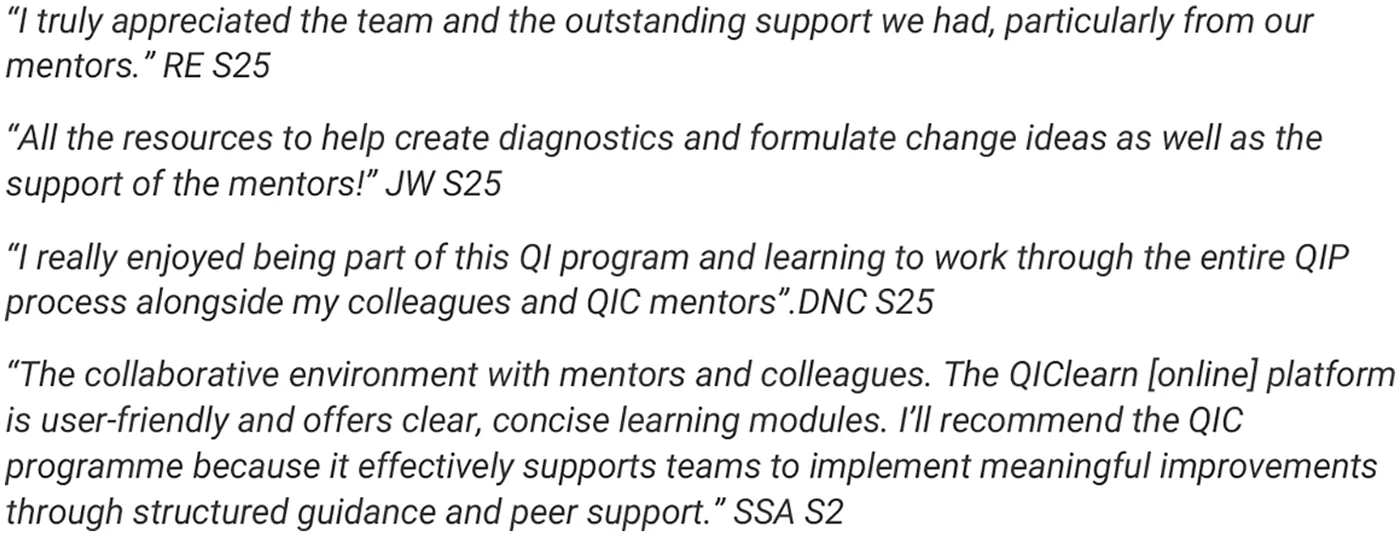
Feedback from learners “ONE thing I loved.”.

### Mentorship and faculty sustainability

This programme has evolved to include both mentorship from experienced QI faculty, but also near-peer mentorship i.e., those who have recently faced similar challenges and who are also “frontline clinicians”. The benefits of peer support and learning within each learning set has been clear from the start, but the concept of developing recent QI Change Champions as MITs (Mentors in Training) has grown over the last five years and we have recent supporting evidence to demonstrate the benefits for building QI capacity in the system as well as programme sustainability through faculty development.

While there are communities of practice for QI, most notably the Institute for Healthcare Improvement (IHI) Quality Improvement community and Open school Chapters (16), mentoring as part of medical education in QI is less well established. The IHI offer practice modules with activities designed to offer experiential learning, but there is no natural progression to becoming a QI mentor and costly coaching courses are aimed at more senior healthcare professionals. NHS QI coaching courses aimed at staff in substantive roles require 5–8 days study leave. This is despite mentoring forming an important component of development in medical leadership and management. Within the literature there are examples of the impact of mentoring on implementing QI (20–24), with some postgraduate training programmes offering a structured mentorship model whilst learning on a QI project (20). Previous studies show that co-learning is feasible, however these have often focused on existing faculty being taught alongside residents or junior staff (21–24). Literature describing or evaluating how to identify and develop effective mentors from resident doctors in QI specifically is lacking. Our approach to faculty development for QI education has not yet been described in the literature despite faculty development being well established within skills-based courses (25), where individuals are identified by existing course faculty to be considered as having instructor potential and recommended for further training. Identification of mentors for experiential learning is often through more ad-hoc self-selection or through matching schemes. Our approach offers a unique opportunity for “QI Change Champions” to continue their QI journey as a mentor in a community of practice, learning both QI and mentoring skills through experience.

Feedback from mentors highlight the benefits to their own personal development as well as the learners they support (Figure 7).

**Figure 7:**
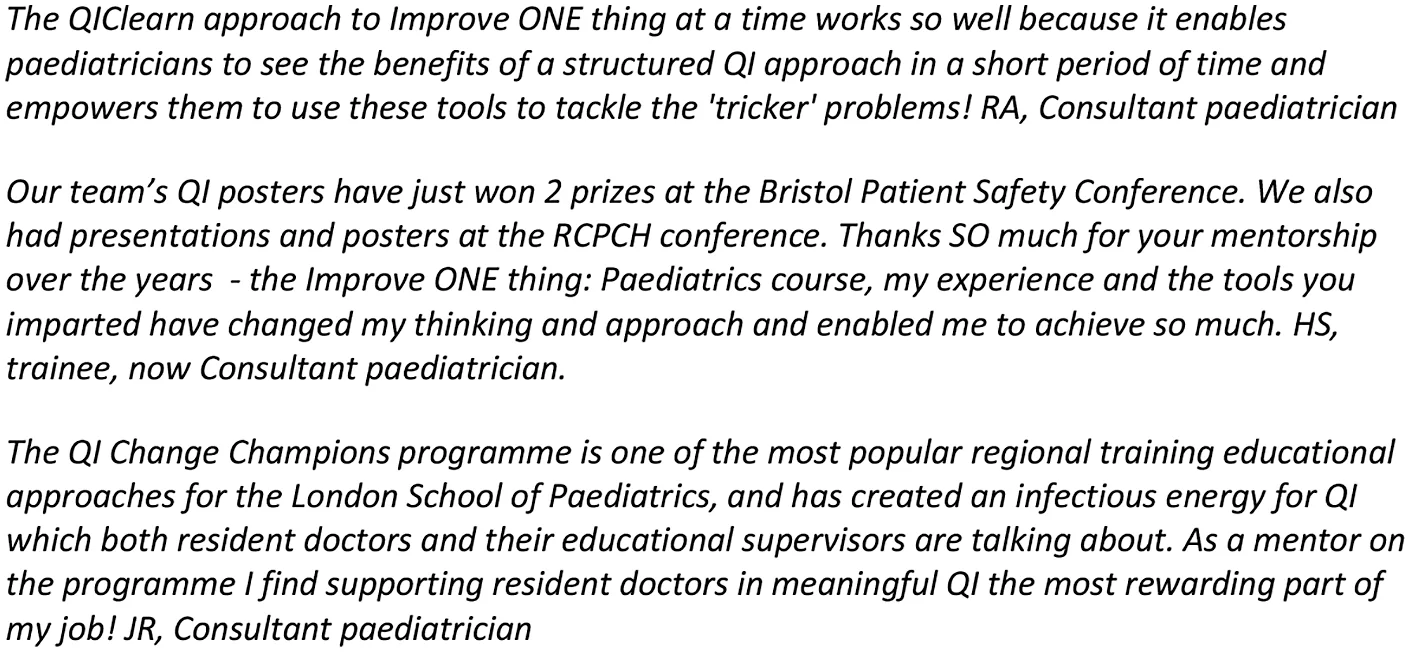
Feedback from mentors.

The table below illustrates the impact the mentors have had in terms of numbers of mentees (learners) supported and the number of cohorts they have engaged with (Figure 8).

**Figure 8:**
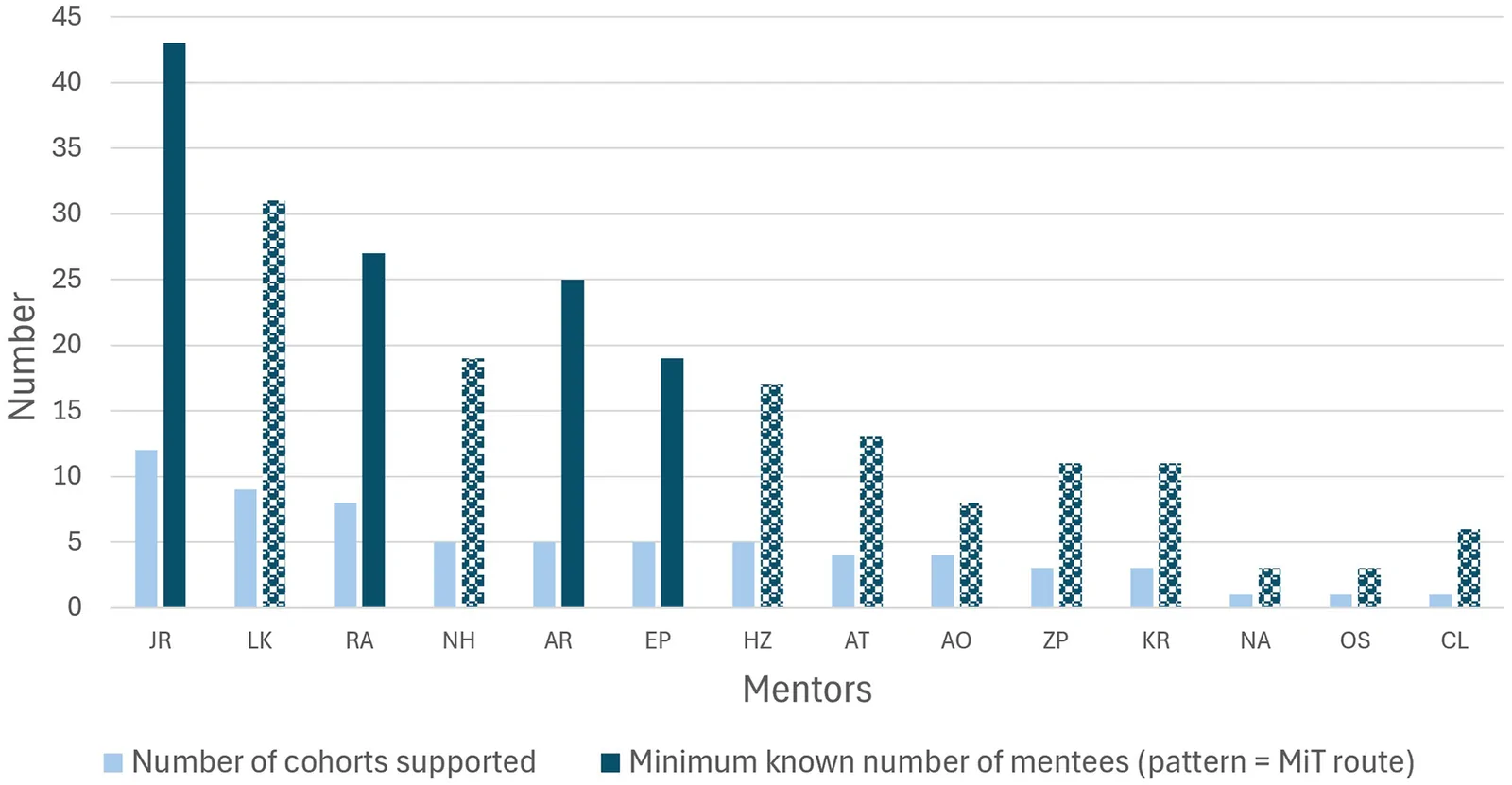
Mentor support of cohorts and mentees (spring 2019- autumn 2025).

The QI methodology approach used to continually develop the programme has also been applied to the mentor-in-training aspect. To ensure that our mentors have the knowledge and experience to best support our learners we have included taught workshops, a separate online space with resources and feedback on the quality of their comments on the online platform and the beginnings of an accreditation process. The mentors themselves are all front-line clinicians so the ‘ask’ of mentors needs to be balanced with their busy clinical roles, whilst providing high quality support to our learners.

Out of 14 mentors sampled in 2025, all reported having taught QI, encouraged others to undertake QI, 13 have mentored on the QIClearn programme (one left the region), 13 have mentored outside of the programme and 11 are currently or have been educational supervisors.

### Multiprofessional working and co-production

The vision of this programme was to reach a point where resident doctors learned with their multi-professional colleagues and patients. Setting up multi-professional learning has many barriers but over the course of 16 iterations of the programme 2 pharmacists, 2 nurses, and 1 dietician have been trained. Originally, the programme included a workshop session on co-production and highlighted resources that could be utilised to include patient and parent voices in the development and co-design of projects. Five parent learners have now completed the programme alongside resident doctors and delivered projects either within a hospital trust or personally to their own child's health needs, with two becoming QI Change Champions, one establishing a children's charity, one becoming a nurse, one becoming a breast-feeding doula, and one becoming a parent advocate on a child safety programme.

The addition of QI to one of the key domains of the RCPCH curriculum, and therefore also a key requirement for a resident doctor's Annual Review of Competency Progression (ARCP), has improved their opportunities for study leave and funding of the programme within the study leave budget of paediatricians. However, one barrier to the MDT learning has been funding AHPs to attend the course, which has become even more challenging as NHS budgets have tightened. The engagement of parent learners, who are offered free places, has also been variable despite careful selection since most have been caring for children with additional health needs. The potential approaches to both multiprofessional working and co-production would be worth exploring further and how best to scale up a programme such as this for multiprofessional teams.

### Impact and QI community development

Over 8 years of delivery, 254 resident doctors have been mentored in London and 150 across the rest of the UK. With 13 resident doctors becoming mentors in training there are now at least 9 faculty which allows mentors to step in and out the programme at points for personal leave. The reach of the projects delivered is expanding. There are 165 posters available on the QIClearn website (26). 50 of these posters have been presented internationally with at least 15 published abstracts and prizes at Patient safety conferences and local Trust awards. Two examples of resident doctors who have spread their work are outlined below: an approach to supporting neurodiverse patients in Emergency Departments that is being adopted within and between regions and a community paediatrician who seeded a national campaign around child poverty. An example of a pharmacist developing the confidence to take her work to wider audiences is also provided (Figure 9).

**Figure 9:**
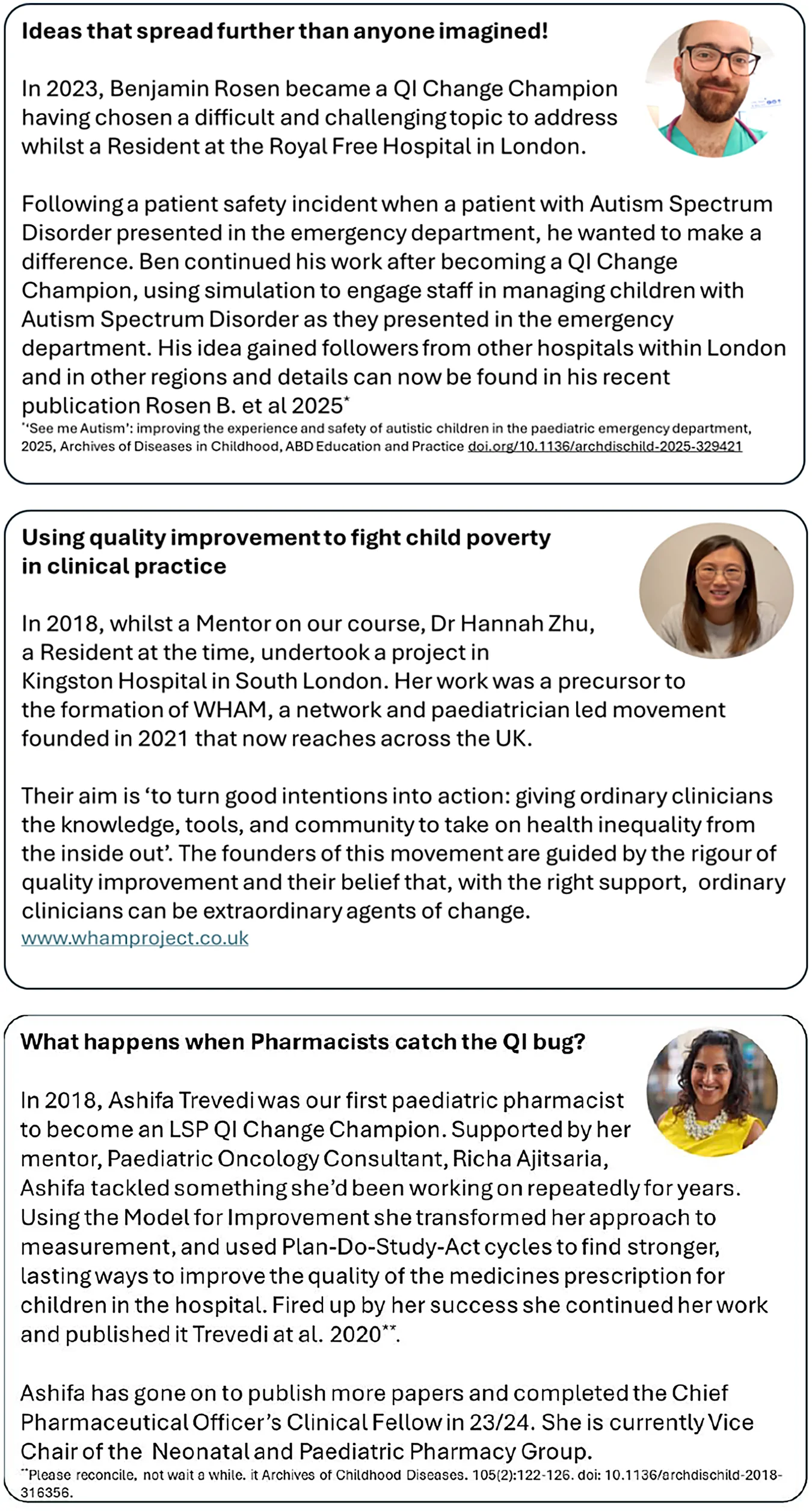
Case examples of the spread of work following the programme.

There is good equity of access for resident doctors as part of LSP. This is in part due to securing recognition within the LSP as a course where there is pre-approved study budget for residents to apply. There is more of a challenge in providing equity across NHS roles.

By building a community of practice (20), learners have been provided with networking opportunities, both on the programme itself through near-peer support and also afterwards. At present 5% of the learners go on to become mentors on the programme. This provides them with structured training to support them to continue their QI journey. A recent survey of QI Change Champions on our programme has shown that around 70% have delivered QI teaching outside of the programme and mentored others in QI activity, with the majority encouraging others in QI and undertaking more local QI activity beyond the programme. The mentors who have been trained on the programme have gone on to complete a further 15 QI projects (excluding those delivered by their mentees).

The building of relationships to further learning and leadership of QI is often under-valued. We are collecting evidence to demonstrate the longer-term benefits of this faculty investment but this needs further research and is an area that could be explored further.

### Transferability and scaling

To facilitate the adoption of this model in other healthcare settings, we have identified the core transferable learning components of the programme versus those elements that were specific and context dependent to our region in London and the NHS (Figure 10).

**Figure 10:**
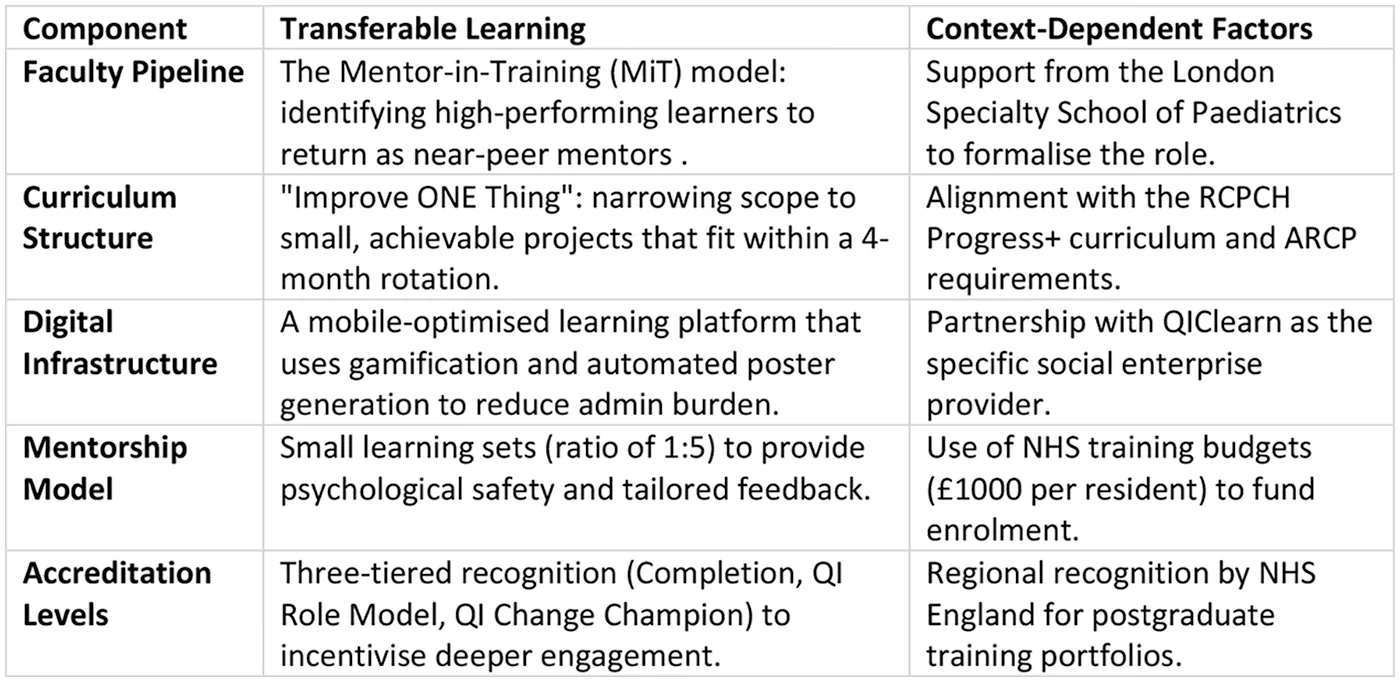
Transferrable learning and context -specific factors.

### Limitations

We recognise the limitations of the data presented as this is not a formal programme evaluation and there could be stronger objective measures of programme impact. We have shared some of our experiences of this programme innovation using survey data from learners and mentors. There is a risk of not reaching all residents in training as there is potentially self-selection bias, with the most motivated residents enrolling. The programme is open to all paediatric resident doctors in postgraduate training in London, and participant recruitment is through bi-annual online application on a first-come first-served basis, with a waiting list process when previous cohorts are over-subscribed. Participants (learners) then consent at enrolment to sharing their feedback survey data to improving programme design. The response rate to the online surveys was consistently over 90% by allocating time at the end of workshops and ensuring completion prior to the issue of certificates required to claim back course fees. Mentor survey response rates were 100% due to individual follow up by faculty. The qualitative analysis of this data is conducted by medical educationalists (NHS clinicians) in the smaller faculty group who are mentors on the programme, (but they are not funded and are independent of QIClearn). This could be seen as a risk of self-reporting bias in response to feedback questionnaires. We have not surveyed a comparable group who have not completed the programme to review their knowledge of QI or experience in conducting meaningful and sustained projects.

## Conclusion

This programme has demonstrated a successful, innovative and sustainable model for embedding essential QI capability into postgraduate training for resident doctors in a region. Over eight years, reaching hundreds of resident doctors, we have demonstrated QI in practice by constantly reflecting, learning and adapting our approach. By recognising what the barriers are to good QI work, this community of learners have been able to adapt more quickly and be agile in their approach to traditional failure.

Importantly, our experience has shown that effective QI education requires more than teaching improvement methodology alone. By combining experiential learning through local QI projects with structured mentorship and participation in a supportive community of practice, learners have developed both the technical skills of QI and the longer-term habits of an improver. Working on projects within their own clinical environments has reinforced the message that QI is a fundamental component of professional practice rather than an additional educational requirement. This aligns with Reed et al.'s description of improvement science as a discipline concerned not only with whether change works, but also how improvement can be achieved, sustained and spread within complex healthcare systems (27). It also reflects the work of Mannion and Davies (28), who highlight the importance of organisational culture, relationships and professional sub-cultures in shaping the success of quality improvement efforts. By creating opportunities for collaboration, peer learning and shared ownership of improvement, QI education programmes may influence both individual capability and the wider culture in which improvement takes place.

Mentorship has emerged as a critical factor in learner success, helping learners to navigate common challenges, apply QI methodology appropriately, and sustain momentum despite the competing demands of postgraduate training. As this programme has taken place in real-time alongside clinical training, we have navigated the challenges of time-constraints and study leave and successfully moved from QI education as a tick-box exercise to showcasing meaningful QI projects in the international arena.

We recognised early on that sustainable QI education depends on development of a faculty development pipeline. Our novel Mentor in Training model has enabled us to both expand our regional QI capacity and develop the next generation of clinical leaders and educators. We have built on the growing field of evidence for near-peer mentors in medical education and developed a real community of practice to support our learners. Early findings suggest that many learners go on to teach, mentor and lead QI activity, indicating that the programme's impact may extend beyond individual projects to influence wider organisational improvement capability. We have some evidence and hypothesise that there are long term benefits to building this community, but this is an area that needs to be explored further in the future.

Our focus now is on future work to help us understand how “to improve the way we improve”. This work needs to focus on scaling up the multiprofessional opportunities and learning within the programme and using our experiences to address financial barriers that hinder wider participation across the workforce. We also have an opportunity for ongoing research and evaluation into the longer term outcomes of the programme in leadership development and sustained QI engagement within the health service. Future work should also explore how this model can be scaled across different professions, specialties and healthcare settings, and how greater patient and public involvement can be embedded within improvement education. These questions align closely with the future research agenda proposed by Reed et al. (2025), particularly around understanding how improvement capability is developed, sustained and spread across healthcare systems.

Our experience in running this programme offers a transferrable framework for any educational body seeking to move beyond theory-based teaching to cultivating a genuine, sustained culture of improvement.

### Literature review

In preparation for submitting this paper the authors conducted an advanced PubMED search using the search phrases “Quality Improvement” AND “Mentoring”. Over 300 papers were identified but the majority were case studies or abstracts. Those referenced in this paper were either seminal papers known to faculty with QI experience, or from the literature search and subsequent references.

## Data Availability

The raw data supporting the conclusions of this article will be made available by the authors, without undue reservation.
